# Applying the lessons of maternal mortality reduction to global emergency health

**DOI:** 10.2471/BLT.14.146571

**Published:** 2015-03-16

**Authors:** Emilie J Calvello, Alexander P Skog, Andrea G Tenner, Lee A Wallis

**Affiliations:** a Department of Emergency Medicine, University of Maryland, 110 South Paca Street, Sixth Floor, Suite 200, Baltimore, Maryland, 21201, United States of America (USA).; b Department of Emergency Medicine, University of California San Francisco, San Francisco, USA.; c Division of Emergency Medicine, University of Cape Town, Cape Town, South Africa.

## Abstract

Over the last few decades, maternal health has been a major focus of the international community and this has resulted in a substantial decrease in maternal mortality globally. Although, compared with maternal illness, medical and surgical emergencies account for far more morbidity and mortality, there has been less focus on global efforts to improve comprehensive emergency systems. The thoughtful and specific application of the concepts used in the effort to decrease maternal mortality could lead to major improvements in global emergency health services. The so-called three-delay model that was developed for maternal mortality can be adapted to emergency service delivery. Adaptation of evaluation frameworks to include emergency sentinel conditions could allow effective monitoring of emergency facilities and further policy development. Future global emergency health efforts may benefit from incorporating strategies for the planning and evaluation of high-impact interventions.

## Résumé

Au cours des dernières décennies, la santé maternelle a figuré en bonne place dans les priorités de la communauté internationale et cela s'est traduit par une baisse considérable de la mortalité maternelle au niveau mondial. Or, même si la morbidité et la mortalité liées aux urgences médicales et chirurgicales sont bien plus élevées comparativement à celles associées aux pathologies maternelles, les efforts menés au niveau mondial pour améliorer les systèmes de soins d'urgence dans leur intégralité attirent beaucoup moins d'attention. Une application réfléchie et spécifique des concepts employés dans l'effort de réduction de la mortalité maternelle pourrait entraîner des améliorations notables au sein des services de santé d'urgence au niveau mondial. Le modèle dit « des trois retards », conçu pour la mortalité maternelle, peut être transposé à la prestation des soins d'urgence. L'adaptation des cadres d'évaluation pour y inclure des critères-sentinelles évocateurs des cas d'urgence vitale pourraient permettre une surveillance efficace des centres d'urgences et la conception de politiques complémentaires. Les futurs efforts consacrés aux systèmes de soins d'urgence au niveau mondial pourraient également bénéficier de l'intégration de stratégies pour la planification et l'évaluation d'interventions à fort impact.

## Resumen

A lo largo de las últimas décadas, la salud materna ha sido un foco importante de la comunidad internacional y esto ha llevado a una disminución considerable de la mortalidad materna a nivel mundial. Aunque, en comparación con las enfermedades de la madre, las emergencias médicas y quirúrgicas son una causa mucho más importante de morbilidad y mortalidad, se ha puesto menos atención en los esfuerzos mundiales para mejorar los sistemas integrales de emergencia. La aplicación profunda y específica de los conceptos utilizados en el intento de disminuir la mortalidad materna puede llevar a mejoras importantes de los servicios sanitarios de emergencia mundiales. El denominado modelo de tres retrasos que se desarrolló para la mortalidad materna se puede adaptar a la prestación de servicios de emergencia. La adaptación de los marcos de evaluación para incluir condiciones centinela de emergencia podría permitir una supervisión efectiva de las instalaciones de emergencia y la elaboración de políticas adicionales. Los esfuerzos futuros en la sanidad de emergencia mundial podrían beneficiarse de la incorporación de estrategias para la planificación y evaluación de intervenciones de gran impacto.

## ملخص

على مدى العقود القليلة السابقة، حظيت صحة الأمومة بتركيز كبير من المجتمع الدولي مما أدى إلى انخفاض ملحوظ في وفيات الأمهات عالمياً. على الرغم من أن الحالات الطبية والجراحية الطارئة هي المسؤولة عن العديد من حالات المراضة والوفيات، والتي تزيد بنسبة كبيرة عن مثيلاتها الناتجة عن أمراض الأمومة، إلا أن الجهود العالمية قد ركزت بدرجة أقل على تحسين أنظمة الطوارئ الشاملة. قد يؤدي التطبيق المدروس والمحدد للمفاهيم المستخدمة في مساعي تقليل وفيات الأمهات، إلى تحسينات كبيرة في الخدمات الصحية العالمية في حالات الطوارئ. يمكن تكييف ما يُسمى بنموذج التأجيلات الثلاثة، الذي تم تطويره بخصوص وفيات الأمهات، ليتم توظيفه في تقديم خدمات الطوارئ. قد يسمح تكييف أطر التقييم لتتضمن حالات الطوارئ الإنذارية، بإجراء مراقبة فعالة لمرافق الطوارئ ومزيد من التطوير للسياسات. قد تستفيد جهود الصحة العالمية في حالات الطوارئ في المستقبل من دمج الاستراتيجيات للتخطيط للتدخلات شديدة التأثير وتقييمها.

## 摘要

在过去几十年中,孕产妇保健一直是国际社会关注的焦点,这导致了孕产妇死亡率在全球大幅下降。然而,与导致发病率及死亡率大大上升的孕产妇疾病、医疗和外科急诊相比,我们却一直不太关注在全球范围内努力改善综合应急系统。周详、具体地应用在努力降低孕产妇死亡率时所用的理念,可显著改善全球紧急医疗服务。所谓的三种延误模式是为降低孕产妇死亡率而制定的,可适用于提供紧急服务。将评估框架运用于紧急报警条件,能够有效监控应急设施,并且有利于进一步制定政策。在规划和评估具有高影响力的干预措施时采用综合策略,将有益于今后的全球紧急医疗工作。

## Резюме

За последние несколько десятилетий вопрос охраны здоровья матерей был в центре внимания мирового сообщества, что привело к существенному снижению смертности среди матерей во всем мире. Хотя по сравнению с уровнем заболеваемости среди матерей на неотложное состояние и срочные операции приходится гораздо больше случаев заболеваемости и смертности, глобальные усилия были направлены на улучшение работы комплексных систем предоставления неотложной медицинской помощи. Обдуманное и конкретное применение концепций, используемых для снижения уровня смертности среди матерей, может привести к значительным улучшениям в деле оказания неотложной медицинской помощи на мировом уровне. Так называемую модель трех задержек, которую разработали для решения вопроса смертности среди матерей, можно применить и к услугам неотложной медицинской помощи. Адаптация системы оценки с включением в нее экстремальных случаев предоставления неотложной помощи позволит проводить эффективный мониторинг учреждений скорой помощи и совершенствовать соответствующую политику. Включение стратегий планирования и оценки высокоэффективных мероприятий может эффективно использоваться в будущих усилиях по предоставлению неотложной медицинской помощи в мировом масштабе.

## Introduction

Global health initiatives are fuelled by the extent of the associated public health need, the severity of the problem involved and the availability of feasible solutions to that problem. In general, the success of such initiatives depends on the organized, concerted and relentless advocacy of international stakeholders – to inspire continued dedication during an often long campaign. The global effort to reduce maternal mortality has benefited from such advocacy, as demonstrated by the progress made towards the achievement of Millennium Development Goal 5 – i.e. towards a 75% reduction of maternal mortality, from its 1990 level, by 2015.^1^^,^^2^ The global community’s approach to improvement in maternal mortality may be applied to other high-impact public health issues, including the delivery of all emergency services.

Compared with maternal illness, medical and surgical emergencies account for far more morbidity and mortality. However, efforts to improve comprehensive emergency systems globally have not achieved as much attention as the improvement of maternal health. Traditionally, attempts to improve the management of emergencies in low- and middle-income countries have been focused on the vertical delivery of health services such as trauma care or responses to obstetric emergencies.^3^ Questions have been raised about the adaptability and flexibility of emergency systems designed around vertical delivery models. For example, it is unclear whether such systems facilitate an adequate response to the new and evolving needs of the communities to be served.^4^ More recently, efforts to develop and improve emergency systems in low- and middle-income countries have included limited horizontal approaches.^5^^–^^7^ Many of these efforts have focused on improving the care provided by ambulance services or other out-of-hospital care, formalizing training for care providers, improved transportation infrastructures and vehicles or the strengthening of public policy.^5^^,^^8^ Despite these inroads into the construction of horizontal emergency systems, there has been scant investigation of effective integrated packages of emergency services or of community engagement to strengthen emergency care. There has been insufficient dialogue on the design of an effective framework to identify, understand and improve areas of weakness in the general emergency systems of low- and middle-income countries.

The right to health has been endorsed by multiple international treaties and national constitutions.^9^^–^^11^ In most low- and middle-income countries, access to good emergency services during a patient’s greatest time of need remains a frequently overlooked but essential element of that right. The far-reaching effects of insufficient emergency systems and health care are particularly apparent in the context of the Ebola virus outbreak in west Africa.^12^ Despite the relative paucity of relevant literature on the building of good emergency systems, it has been estimated that integrated prehospital and in-hospital emergency systems could address 35–46% of morbidity and mortality in low- and middle-income countries.^3^ The burden of emergencies – like the burden of maternal illness – falls largely on low- and middle-income countries.^13^ Many of the lessons learnt from efforts to reach Millennium Development Goal 5 in low- and middle-income countries are transferrable to the critical barriers in the development of effective emergency systems. These lessons include the unified conceptual framework required to achieve a holistic understanding of the large morbidity and mortality burdens caused by emergencies of all types – infectious disease, noncommunicable disease and trauma. Such a framework is also a key element in the evaluation of impacts and the direction of any proposed interventions. To describe a potential framework, we used standardized terms that refer to certain aspects of emergency systems, services and care (Box 1).^3^^,^^14^

Box 1 **Definitions of terms used in emergency health**

**Emergency systems**

All organizations, institutions and resources whose primary purpose is to promote, restore and/or maintain health in medical and/or surgical emergencies

**Emergency services**

The sum of all efforts to deliver effective health action in response to extreme risk under intense time pressure, including interventions at both the population level and the individual level

**Emergency care**

The subset of emergency services focused on delivery of curative interventions targeted at severe clinical cases – the prime tool for addressing emergent health conditions that present sudden or unexpected threats and thus a critical output of the overall health system

## The three-delay model

**Fig. 1:**
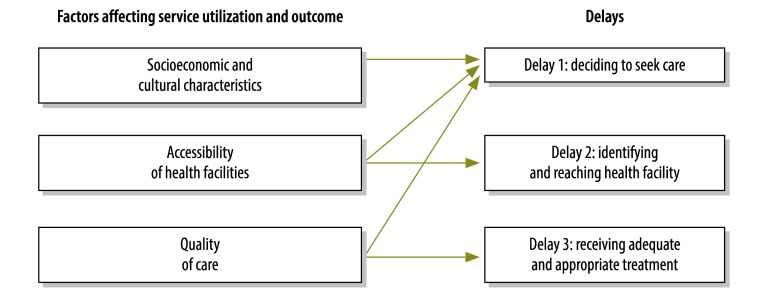
The three-delay model in emergency care

The recent decrease seen in maternal mortality is a product of interdisciplinary efforts that used multiple approaches to increase service availability and remove financial barriers to care.^15^ An early model provided an invaluable framework for understanding not only the factors contributing to the mortality resulting from obstetric emergencies but also the initiatives that may have most potential impact.^16^ A later model focused on the three main factors that affected the outcome of emergency presentation during pregnancy. These factors were defined, chronologically, as the lengths of the delays in: (i) the decision to access care, (ii) the identification of – and transport to – a medical facility, and (iii) the receipt of adequate and appropriate treatment.^17^ Socioeconomic and cultural factors, accessibility of facilities and quality of care may independently affect the lengths of these three delays (Fig. 1). This so-called three-delay model illustrated that maternal mortality was not due solely to a lack of economic and human resources but was a product of numerous interwoven factors. A poor patient outcome is likely to result if any of these factors contribute to an undue delay. For example, an inability to recognize an emergency may extend the delay in the decision to seek care. While the ability of the patient or a caregiver to recognize an emergency is partially dependent upon the patient’s or caregiver’s level of education, studies have shown that true obstetric emergencies may not be perceived as emergencies in areas where they commonly occur.^18^^,^^19^ Additionally, in various cultures, women’s status can affect both the ability of women to decide to seek care and their subsequent ability to reach care.^20^^,^^21^

### Transferability of the model

The established definition of general emergency services – as all efforts to provide services, in time-sensitive conditions, to patients and populations under extreme risk – may easily be extended to obstetric emergencies as conceptualized in the three-delay model.^3^ General emergency services are not restricted to the provision of medical interventions but also require timely action.^3^ In the three-delay model, the contributors to delays are not specific to emergencies during pregnancy but can be applied to emergencies in general (Fig. 1). The barriers posed by transport access, distance to care and gender-specific differences in cultural status, for example, are relevant to all emergency services.^22^ Similarly, the barriers posed by distance from health facility and the perception of the quality of services – which have been shown to increase the time it takes for a sick pregnant mother to access care^17^ – are common targets of programmes to improve the delivery of emergency service in general.^23^^,^^24^

**Fig. 2:**
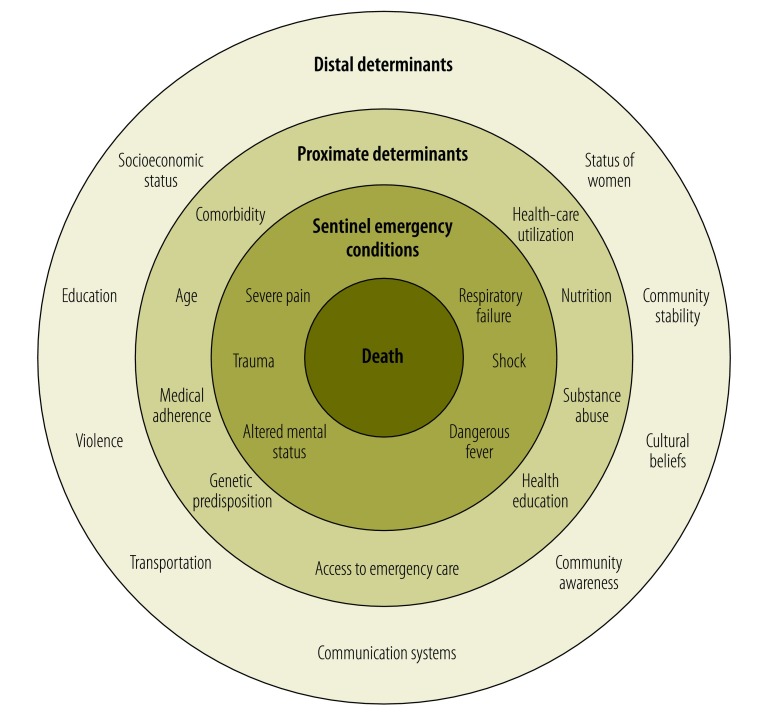
Emergency sentinel conditions and their determinants

Note: Severe pain includes severe headache, chest or abdominal pain.

Efforts to reduce maternal mortality have benefited from the relatively small number of etiologies that contribute to most such mortality – i.e. haemorrhage, sepsis, unsafe abortion, pre-eclampsia, eclampsia and prolonged obstructed labour – and the corresponding effective treatments that are available.^25^ It has been estimated that 74% of maternal mortality could be averted if all women received appropriate emergency obstetric care.^26^ Although general emergency health involves a vast array of etiologies, there are relatively few conditions that, if left untreated, frequently and rapidly progress to death (Fig. 2).^3^ In November 2013, at the African Federation of Emergency Medicine Consensus Conference in Cape Town, South Africa, six emergency sentinel conditions – shock, respiratory failure, dangerous fever, severe pain, trauma and altered mental status – were endorsed by 150 physicians as the basis of an effective organizational framework for emergency care.^27^ Shock, respiratory failure, trauma and altered mental status are commonly understood clinical syndromes. Severe pain encompasses conditions such as cardiac ischaemia, acute abdominal pathologies and causes of headache that are medical emergencies.^15^ Dangerous – i.e. life-threatening – fever has many possible etiologies but is frequently the result of infections, environmental conditions, endocrine abnormalities or toxins.^28^ As with the small number of conditions that cause most maternal mortality, there are critical time points when appropriate interventions may prevent the progression of each sentinel emergency condition.

### Critical time points

#### Seeking care

Effective emergency care is dependent upon the patient’s or caregiver’s ability to recognize that an abnormal condition exists, that the condition has a level of severity warranting intervention and that an intervention is available to treat the condition.^17^ Any slowness in the recognition of a potentially severe condition will decrease the likelihood that appropriate care will be provided in a timely and effective fashion. Cost appears to be a less important barrier to utilization than other factors, especially when an emergent condition is present.^17^ The patient’s or caregiver’s perception of the quality of care that the patient will receive does seem to have a strong effect on their decision to seek care.^29^ These findings have been recently validated, for all types of emergencies, among communities in rural Kenya and Zambia.^30^

#### Reaching care

The delay in identifying and reaching a medical facility is dependent upon the planning and organization of prehospital emergency services in the setting involved. Effective prehospital care – even in low-resource settings – improves survival by decreasing the time to treatment.^6^^,^^7^ Community-based first responders can reduce mortality and morbidity substantially, especially among trauma cases. In northern Iraq, for example, the mortality rates from penetrating trauma and land-mine injuries declined from 91% to 15% and from 28% to 9%, respectively, after community-based first responders were trained to provide field care for such traumas and to cooperate with paramedics when needed.^6^ If care delays are to be minimized, the individuals who provide prehospital services need to be able to identify the level of care that a patient requires and to take the patient directly to the nearest facility that offers that level of care.^31^

#### Receiving appropriate care

**Fig. 3:**
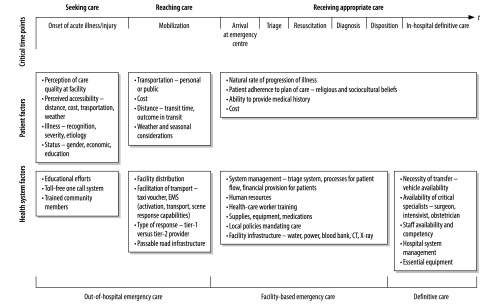
Conceptual framework for critical time points in emergency care

The delay in the receipt of appropriate care – after the patient has reached a health facility – may be broken down into three parts: the delay in the provision of appropriate care at the initial facility, the delay in the patient’s transfer to another facility for definitive care – if needed, and the delay in the provision of appropriate care at the second facility – if needed (Fig. 3).^32^ Delay at any of these time points has been shown to worsen patient outcome.^33^^–^^35^ For cases of sepsis, for example, rapid triage at a health-care facility and early therapy – which should be possible even in low-resource settings – can reduce mortality substantially.^36^ In rural Malawi, paediatric mortality was decreased by providing resuscitation in the emergency department instead of in the wards, formalizing the triage process and decreasing the time it took for patients to see senior-level providers.^37^

CT: computed tomography; EMS; emergency medical services.

Note: Tier-1 providers are community-based voluntary or professional first responders whereas tier-2 providers are the paramedics, emergency medical technicians and ambulance technicians who provide specialized prehospital care.

### Monitoring provision

Each of the delays considered in the three-delay model is determined by a multiplicity of factors. Each must therefore be assessed by gauging broad indicators of outcome rather than by considering service utilization alone. The danger of monitoring the provision of emergency care simply in terms of service utilization is illustrated by the results of a recent prospective cohort study in El Salvador.^38^ In this study, the rates of sepsis and infectious mortality in children with acute leukaemia who were being treated for fever were investigated. Although all of the study children were being treated at a single hospital, those who had had to travel relatively long distances to reach the hospital and those with relatively low household incomes had taken longer to receive antibiotics and had poorer outcomes than the other children. Additionally, the time taken in deciding to seek care for a sick child was found to be greater when the child’s mother was illiterate.^38^

The assessment of broad indicators rather than service utilization was recommended by the World Health Organization (WHO). *Monitoring emergency obstetric care: a handbook*^39^ identifies broad indicators for emergency obstetric care and defines acceptable levels for each indicator as appropriate delivery targets. For example, one indicator is the minimum of five emergency obstetric-care facilities for every 500 000 people, with at least one facility providing comprehensive emergency obstetric services. Systems’ capability can be assessed by using the acceptable levels as benchmarks and checklists to assess the factors that contribute to each delay of the three-delay model.^15^

While indicators can be used to evaluate an entire system’s performance, the care being provided for each type of major emergency at the facility level also needs to be assessed. *Monitoring emergency obstetric care: a handbook* describes so-called signal functions – i.e. life-saving services – for each major cause of maternal death. These functions can be used to assess a particular facility’s ability to prevent delays in the receipt of appropriate emergency care, after a patient has reached a health facility. The successful implementation of signal functions indicates the existence of a functional system of emergency care. Each such function represents a culmination of knowledge, interventions and supplies. Performance can therefore be assessed by investigation of such functions, without the need to assess the individual components of each critical intervention. For example, researchers who observe the effective administration of parenteral antibiotics in a facility may reasonably conclude that the facility has personnel who can choose appropriate antibiotics, give drugs intravenously and can administer the relevant tubing, catheters and medication. If any component of the signal function is absent, then that function cannot be accomplished and the system of care is deficient. This concept is particularly applicable to emergency care, where a concatenation of many events must often occur to produce the desired function. Checklists to help assess the capabilities of individual facilities in completing each signal function for maternal emergencies have already been developed and successfully deployed.^40^ The African Federation for Emergency Medicine has expanded the concept of generalized emergency sentinel conditions to include signal functions and their associated required supplies.^27^ Validation of the resultant emergency-care assessment tool for health facilities is currently underway.

### Lessons learnt

The recent focus on assuring the delivery of effective obstetric emergency care to reduce maternal mortality has led to the development of an intellectual framework that is largely applicable to global emergency health in its broadest terms. Although recent developments in obstetric care have much in common with potential developments in general emergency care, the inherent differences need to be appreciated. Specifically, obstetric care is centred on a physiological event that most often concludes with few complications and a new life. When they do arise, complications are usually limited to the time of gestation or birth. In contrast, emergency care provides essential care for pathological processes – including acute exacerbations of noncommunicable disease, acute infections and trauma – that can happen at any point in an individual’s life.^41^

The natural time constraint of obstetric emergency care has often allowed interventional packages based on relatively simple solutions to be successful – although a more comprehensive functional system may be needed to have scalable effectiveness.^42^ Notably, a recent large multicountry survey by WHO revealed a substantial mismatch between good health outcomes and high coverage of essential health services.^43^ The mismatch was thought to be attributable to a shortage of comprehensive emergency care for women. In the absence of a comprehensive patient-centred approach, provision of a single element of care is unlikely to improve mortality or morbidity. Together, haemorrhage, sepsis and hypertensive emergencies cause 52% of maternal mortality but these are not just pregnancy-related issues as they can lead to mortality via the same pathways as emergency sentinel conditions.^25^ Consequently, interventions to treat these emergencies and others can strengthen entire emergency systems and lead to many improvements other than the expected reduction of maternal mortality.^43^ A broad emergency system that provides universal access to life-saving interventions is able to treat emergencies in pregnancy as well as trauma and medical emergencies.

We will need substantial infrastructural changes to emergency systems if each of the three main delays in emergency care is to be minimized. However, the multiple changes needed for a complete overhaul of emergency systems may not be possible to implement simultaneously, particularly in low-resource settings. Recent efforts to offer a roadmap to overcome neonatal mortality in low- and middle-income countries have focused on a few key strategies. The same strategies could be applied more broadly to emergency systems – by advocating for universal health care, making emergency services free to all, developing a system that provides a basic level of emergency care at community level, and developing strong monitoring programmes to assure that key emergency services are being delivered at health facilities.^44^ Such strategies could be pursued with those targeted practical interventions that have been shown to be markedly effective when focused on critical time points in the chain of survival.^6^^,^^16^^,^^36^ The most cost–effective initiatives tend to be those targeted at the delays in the decision to access care and in the identification of – and transport to – a medical facility. For example, education on the recognition of emergencies and how to access the appropriate level of care can be particularly effective but relatively inexpensive. Investment in the training of community members to assist with emergency identification and the transport of patients to appropriate care has been shown to significantly decrease mortality.^6^^,^^7^ The staff in the more basic health facilities can be trained to provide interventions that can sustain a patient’s life until the patient reaches a facility where definitive care is available.^23^^,^^45^^,^^46^ It is a common misconception that substantial investment in infrastructure at a health-care facility is required to accelerate access to appropriate care. In fact, the implementation of standardized emergency training courses – e.g. WHO’s Emergency triage assessment and treatment training course – can lead to substantial reductions in mortality without any major investment in material infrastructure.^47^

## Conclusion

While prevention remains critical, treatment – within the context of a patient-centred supportive system – will be needed if we are to achieve large sustained reductions in death and disability resulting from emergency presentations. As with maternal health, emergency care requires not only that the patient or caregiver recognizes that a life-threatening or life-changing condition is occurring, and that there is a need to seek care, but also that timely access to adequate care is available. Given the unpredictable nature of health emergencies, there are few quick fixes. However, strong emergency systems can prevent delays at critical time points. Such systems do not require massive resource allocation but rather a cost-effective, informed approach that emphasizes the proven life-saving interventions that are appropriate to the context. Improving access to emergency care, by minimizing the three main types of delay in the delivery of such care, has the potential to reduce mortality in every field, system and population.
